# Biomechanical comparison shows increased stability of an arthroscopic subscapular sling procedure compared to an open Latarjet reconstruction for anterior shoulder instability in specimens with major glenoid bone defect

**DOI:** 10.1002/jeo2.70015

**Published:** 2024-09-23

**Authors:** Terje Vagstad, Jan Arild Klungsøyr, Christian Bjerknes, Petter Klungsøyr, Aleksander Skrede, Andreas Dalen, Jon Olav Drogset, Tor Åge Myklebust, Erland Hermansen

**Affiliations:** ^1^ Department of ICT and Natural Sciences Norwegian University of Science and Technology (NTNU) Ålesund Norway; ^2^ Orthopedic Department Møre and Romsdal Hospital Trust Ålesund Norway; ^3^ Department of Neuromedicine and Movement Science Norwegian University of Science and Technology (NTNU) Trondheim Norway; ^4^ Hofseth BioCare Ålesund Norway; ^5^ Orthopedic Department Trondheim University Hospital Trondheim Norway; ^6^ Department of Research and Innovation Møre and Romsdal Hospital Trust Ålesund Norway; ^7^ Department of Health Sciences Norwegian University of Science and Technology (NTNU) Ålesund Norway

**Keywords:** anterior glenohumeral instability, arthroscopic subscapular sling procedure, biomechanical study, Latarjet procedure, major glenoid bone defect

## Abstract

**Purpose:**

Recurrent anterior glenohumeral instability (RASI) is commonly treated with arthroscopic techniques, though their effectiveness in providing stability may diminish in cases of critical glenoid bone loss. This study aimed to compare the stability outcomes and range of motion (ROM) associated with an arthroscopic subscapular sling procedure (SSP), first introduced in 2015.

**Methods:**

Sixteen fresh‐frozen human cadaveric shoulder specimens were biomechanically evaluated in four conditions: native, injured, post‐SSP and post‐LP. Glenohumeral translations were measured under anterior, anteroinferior and inferior loading, while external rotation ROM was assessed in neutral and abducted positions. Testing was conducted using a robotic system for precise force and torque application. Specimens were prepared with a 20% glenoid bone defect and subjected to stability testing sequentially.

**Results:**

The SSP significantly reduced glenohumeral translations compared to LP, particularly under anterior loading in neutral (*p* < 0.001), external rotation (*p* = 0.007) and abduction (*p* < 0.001) positions. Although the SSP demonstrated superior stability in these key positions, it did not consistently outperform the LP across all scenarios, as stability was similar between the two in the abducted and rotated position under anterior loading (*p* = 0.379). Under anteroinferior loading, the SSP showed comparatively better stability at neutral (*p* = 0.003) and abduction (*p* < 0.001), whereas the LP led to greater anteroinferior translations in these same positions (*p* = 0.002 and *p* = 0.014, respectively). The SSP outperformed the LP under inferior loading in neutral (*p* = 0.005) and abduction (*p* = 0.02) positions, though it did not fully restore stability to native shoulder levels. The SSP maintained ROM similar to native shoulders. LP allowed greater ROM, potentially compromising stability.

**Conclusion:**

The SSP provided greater stability than the open Latarjet in most positions and did not limit ROM, suggesting it could be a viable, less invasive option for managing shoulder instability.

**Level of Evidence:**

Not applicable.

**Abbreviations**
LP: Latarjet procedure
RASI: recurrent anterior shoulder instability
ROM: range of motion
SSP: subscapular sling procedure

## INTRODUCTION

Deciding on the most appropriate treatment for recurrent anterior shoulder instability (RASI) can be challenging. As a first‐line treatment option, nonsurgical management has increasingly fallen out of favour, particularly for the younger patient populations. Each repeat episode of instability may increase the likelihood of future surgical failure [40, 45]. Surgery is commonly recommended for patients with recurrent instability and can be performed by open surgery or arthroscopically. Among the most common procedures are the well‐established Bankart and Latarjet procedures [1]. Traditionally, arthroscopic Bankart has been recommended for patients without significant (≥20%) glenoid bone loss [6]. However, for bone loss in the range of 13.5%–20%, further soft tissue augmentations are recommended [12], such as a remplissage in situations of concurrent off‐track Hill–Sachs lesions [32]. Clinical outcomes may still be unsatisfactory for subcritical glenoid bone loss (13.5%–20%) compared with patients that undergo arthroscopic Bankart with bone loss less than 13.5% [46]. According to some long‐term studies, recurrent instability rates may be as high as 17%–35% 5–10 years after arthroscopic Bankart repair [4, 5, 25, 39]. In cases of bone loss exceeding 20%, coracoid transfer procedures are widely endorsed, with generally favourable clinical outcomes [7, 28, 42]. In employing the Latarjet procedure, the coracoid process with its conjoined tendon is transferred to the glenoid rim, restoring bone loss and the glenoid surface. The conjoined tendon limits translation during abduction and external rotation, termed ‘the sling effect’ [10]. Despite its success, the open Latarjet procedure is associated with comparably high complication rates [13, 31, 46]. In 2007, an arthroscopic approach to the Latarjet procedure was described, with favourable clinical outcomes compared with the open technique. It is, however, considered by many to be a technically challenging procedure with a significant learning curve for the surgeon [14, 15], which holds true even for experienced shoulder surgeons [38]. Nevertheless, critical glenoid bone lesions are commonly considered an absolute indication for open surgery [43]. The arthroscopic Latarjet approach is likely to continue to improve in safety as guided techniques and improved fixation devices are developed [17, 26].

**Figure 1:**
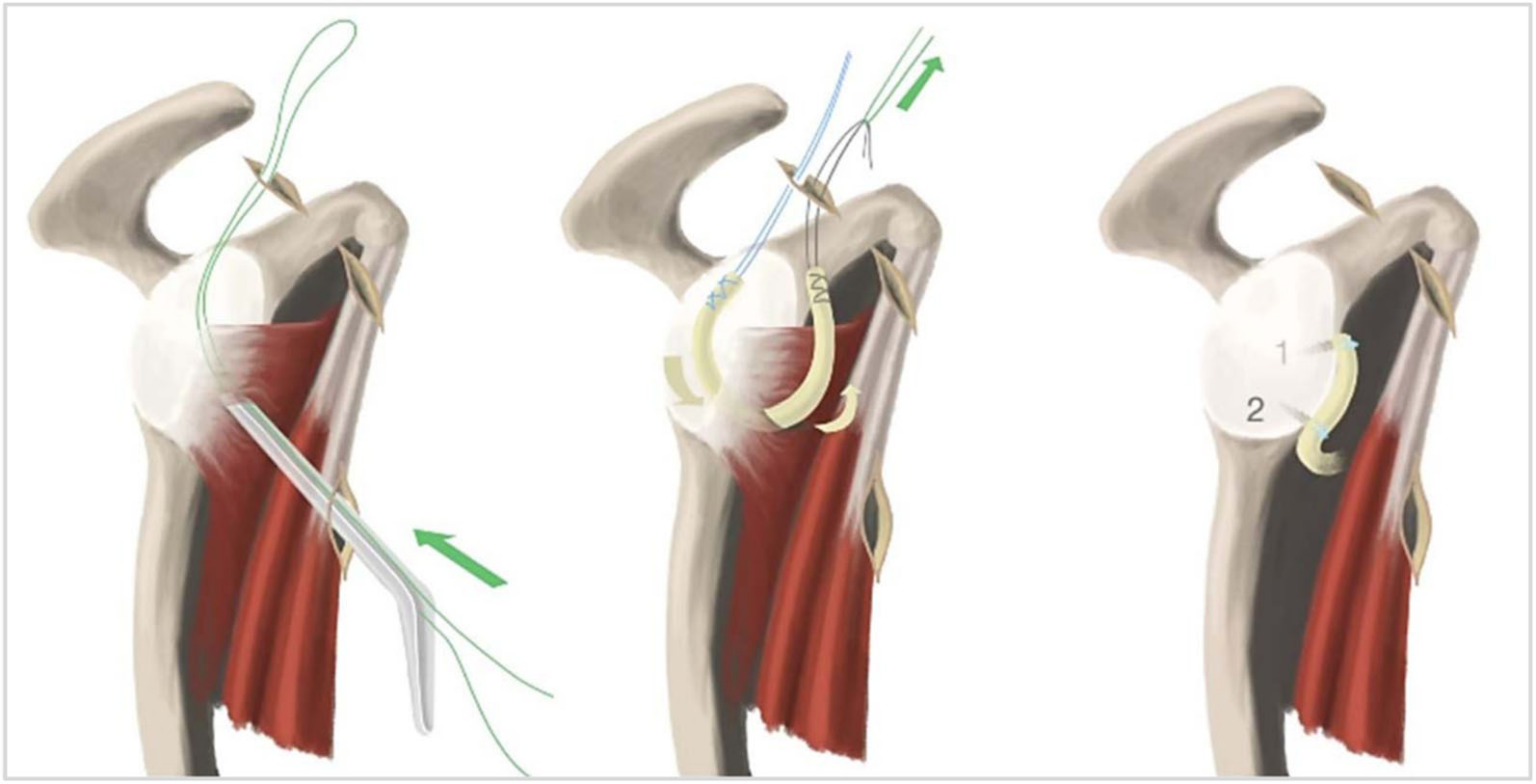
Arthroscopic subscapular sling procedure illustration of a right shoulder. With an incision in the subscapular tendon, a passing suture is introduced through an upper portal into the joint and subsequently exited through a portal at 5 o'clock. After passing the autograft into the joint, it is attached to the anterior glenoid rim with suture anchors.

In pursuit of arthroscopic procedures that may complement the current therapeutic options for recurring glenohumeral instability, the subscapular sling procedure (SSP) was first described in 2015 by Klungsøyr et al. [22]. This procedure adopts the ‘sling phenomenon’ as observed in the Latarjet procedure. In the SSP, an autologous semitendinosus graft encircles the upper part of the subscapularis tendon and is attached to the anterior rim of the glenoid, creating a sling phenomenon which opposes dislocation during external rotation and abduction. The procedure effectively reconstructs the anterior labrum (Figure 1). The sling assists in stabilizing the humeral head in the glenoid fossa, by preventing anterior and antero‐inferior migration of the subscapularis tendon by pulling it posteriorly. Biomechanical studies have demonstrated the feasibility and encouraging stabilizing properties of this procedure [19, 20, 22, 37]. Obtaining a graft is a well‐stablished and safe practice [8, 11]. Concurrently, a small ongoing clinical trial is recently published [21], to determine safety, clinical and radiological outcomes of the SSP and the clinical results from this study. For patients with recurrent and chronic anterior shoulder instability, in which a given degree of subcritical glenoid bone loss complicates choice of established procedures, the SSP may hold promise as a viable alternative that serves to fill the gap between a Bankart procedure and Latarjet procedure. The purpose of the current study was to compare the biomechanical stability of the subscapular sling procedure with an open Latarjet procedure in cadaveric shoulders with 20% glenoid bone loss.

## MATERIALS AND METHODS

A total of 17 fresh‐frozen human cadaveric shoulders without a prior history of injury were collected (Science Care). One shoulder was employed to establish the final testing protocol and was also kept in reserve. In total, 16 cadaveric shoulders were included as samples in this preclinical biomechanical investigation.

A temperature of −23°C was used for the storage of all specimens. Before testing, specimens were thawed for 12 h at room temperature. Ethical approval for this study was obtained from the Regional Committees for Medical and Health Research Ethics in Norway (reference number:2018/2023 REK sør‐øst).

### Specimen preparation

Neutral rotation of the humerus was determined by flexing the elbow to 90° and inserting a K‐wire parallel to the forearm antero‐posteriorly into the humeral shaft, approximately 16 cm below the superior border of the humeral head. Osteotomy of the humerus was performed 5 cm below the K‐wire, providing a means of embedding the humeral end in a brass cylinder by employing a casting resin (RenCast© FC 52/53 Isocyanate/FC 52 Polyol; Huntsman Corp.). The skin, soft tissue and muscles of the distal two‐thirds of the scapula wing were resected. The scapula was cast into a custom‐made box. During shoulder elevation, the scapula tilts posteriorly and rotates both upward and externally, a function referred to as physiological tilting [34]. To simulate physiological tilting, the scapula block was attached to the mounting plate using three threaded rods and tilted 10° forward. This scapular fixation permitted stable and reproducible mounting of specimens during surgery and robot‐assisted testing.

**Figure 2:**
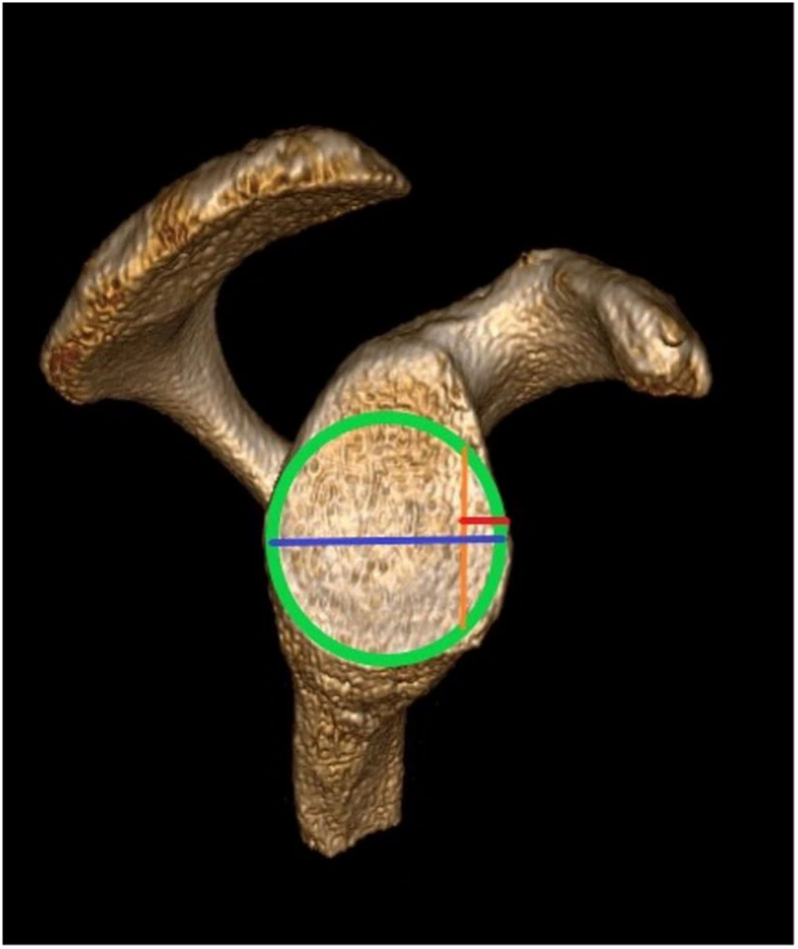
Preoperative CT image in 3D. Diameter of best‐fit circle (green), glenoid width (blue), 20% calculated glenoid bone defect (red/orange). Ref mangler.

A preoperative CT scan and three‐dimensional reconstruction (3D CT) of all the scapulae were obtained to exclude pathology in the joint and tendons and for the planning of the anterior glenoid bone defect [35]. A perfect circle was displayed in the inferior two‐thirds of the glenoid in a sagittal section of the glenoid *en face* view (Figure 2). Using 20% of the circle's diameter, the width of the anterior bone fragment was determined [16, 35].

**Figure 3:**
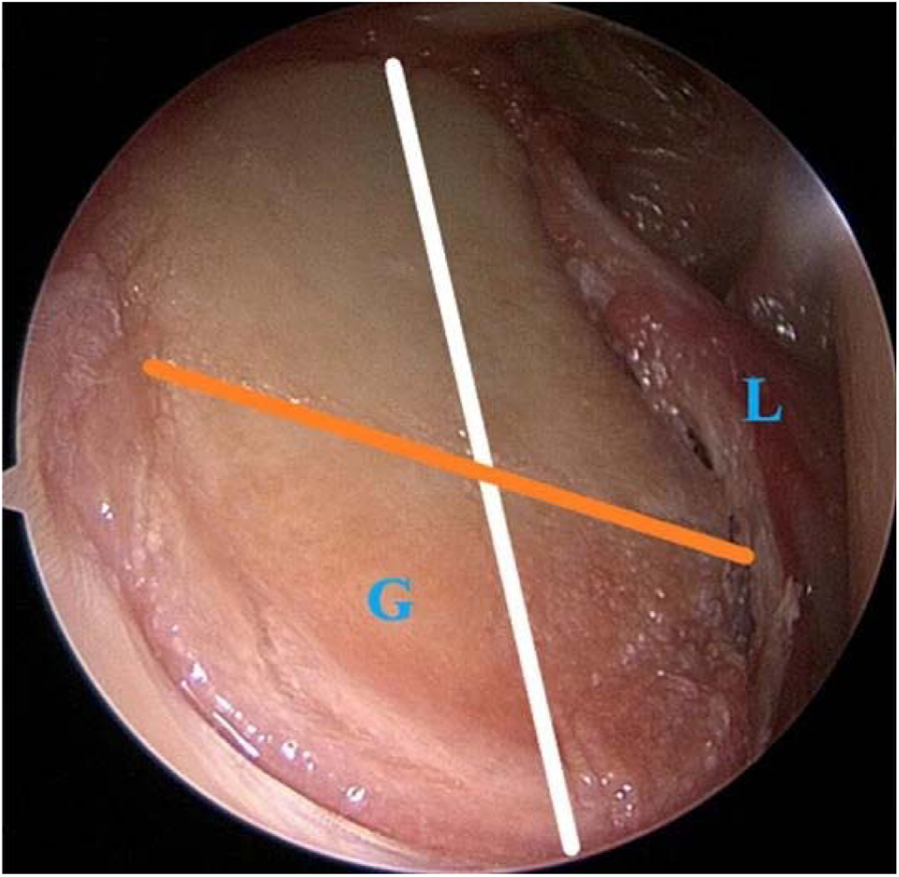
Anterior glenoid resection performed parallel to the white line between 12 and 6 o'clock. Orange line marks the diameter of best‐fit circle.

Anterior and antero‐superior portals were positioned during surgery of the cadaveric shoulders with the assistance of a standard posterior camera portal. The anterior portal was created just above the superior edge of the subscapular tendon. The antero‐superior portal was placed just in front of the anterior edge of the acromion. The glenoid labrum was detached from 2 to 6 o'clock. Anterior glenoid resection was performed parallel to the axis between 6 and 12 o'clock, recreating the clinical phenotype of glenoid bone loss (‘0° osteotomy model’) (Figure 3) [27]. Using chisels with widths ranging from 3 to 7 mm, a precise resection line was established along the anterior rim of the glenoid. The exact amount of resected bone loss was measured using a precision digital caliper with submillimeter accuracy, immediately following the glenoid bone resection from cadaver shoulder samples. This method is validated for its precision and reproducibility, making it suitable for accurately quantifying bone loss in this study. It ensures that the data generated from the 20% bone loss model is consistent and reliable [27].

### Robotic testing protocol

**Figure 4:**
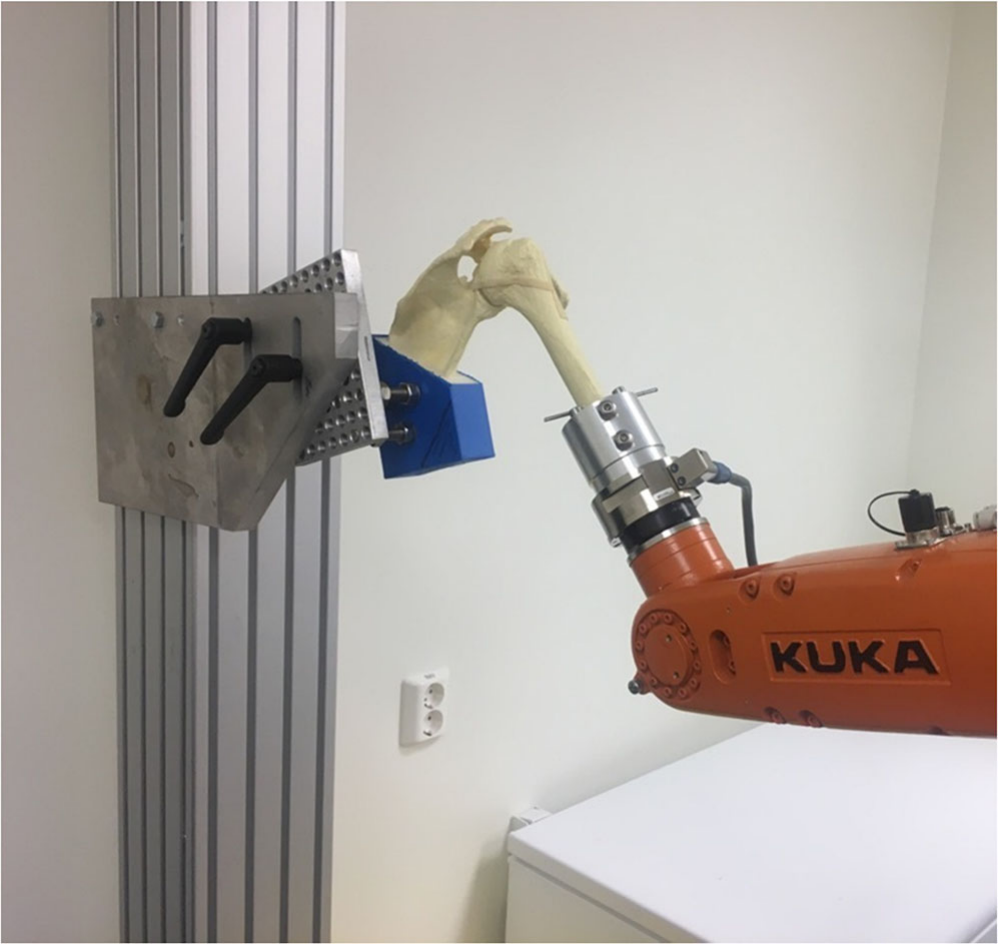
kuka robot with shoulder set up, Biomechanics Lab Ålesund, Norway.

Passive biomechanical stability and range of motion (ROM) of the specimens were tested by an industrial robot (KR 6 R900 sixx; KUKA Roboter GmBH) equipped with a force/torque sensor (FTS) (F/T Sensor Gamma; ATI Industrial Automation) attached to the robot flange. Specimens were mounted firmly in a rig and oriented so that the anatomical axes of the specimen were parallel to the robot coordinate system (Figure 4). The FTS measured three components of both force and torque, which were transformed to the anatomical coordinate system, allowing the robot to perform force/torque‐guided motions of the specimen. The robot Tool‐Center Point (TCP) was defined to be in the humeral head (HH) rotation centre, allowing abduction and external rotation to be modelled as torque‐guided rotations of the TCP and stability (translation) tests to be modelled as force‐guided linear translations of the TCP. Before robotic testing, each specimen was injected with 20 mL of ambient air into the glenohumeral capsule to neutralize the vacuum effect. Arthroscopic procedures were performed without the addition of water. A full overview of the robotic testing protocol is given in a separate publication [33].

At the start of the test protocol, the humeral head was centred in the glenoid cavity by the robot, using the force guidance strategy to achieve 30 N medial force, and 0 N in the anterior and superior directions. Then, stability was measured by the robot in four different poses of the shoulder as in previous studies [19, 20, 37], specifically in (i) 0° abduction and 0° external rotation, (ii) 0° abduction and 60° external rotation, (iii) 60° abduction and 0° external rotation and lastly (iv) 60° abduction and 60° external rotation. In each of these poses, stability was tested in the anterior, inferior and antero‐inferior directions. The force setpoint for translation testing was 30 N in the test direction and 30 N medially.

The normal biomechanical testing positions are 60/60 of glenohumeral abduction and external rotation with a fixed scapula. This position (60/60) represents 90° of shoulder abduction and external rotation when scapula is allowed to move freely, as pointed out in previous biomechanical publications [41].

ROM tests were used to check if the external rotation of the glenohumeral joint was impaired following surgery. Each cadaveric specimen was tested for glenohumeral translation and ROM in four different conditions consequently: (1) native and ventilated, (2) injured, (3) after the arthroscopic SSP and (4) after the open Latarjet procedure. The ROM tests were performed following the translation tests in (i) 0° abduction and 60° external rotation; and (ii) 60° abduction and 60° external rotation. The ROM test was performed by external rotation until a 2 Nm torque was achieved, and the measurement of the ROM test was the angular distance from 0° external rotation to this converged orientation of the humerus.

### Semitendinosus harvest

Semitendinosus grafts were harvested from intact cadaveric human knees (Science Care), accomplished through a small incision on the proximal medial tibia. Grafts were doubled and whip‐stitched. The graft used for the SSP is a regular semitendinosus graft with an average thickness between approx. 6 and 7 mm and length approx. 25 cm.

### SSP

The SSP was performed as previously described [19, 20, 22]. With a blunt dissection, the layer anterior to the subscapularis tendon was cleared. To avoid injury of local neurovascular structures, the lower anterior 5 o'clock portal was created lateral to the conjoined tendon with the aid of an aiming device (Arthrex GmbH), switching stick and halfpipe instruments. The humerus was externally rotated 15° and an incision was made 3 cm below the upper subscapular tendon margin under the guidance of the aiming device, longitudinal to the tendon fibres between the lower and middle third of the subscapular tendon. The incision measured approximately 2 cm in width and extended medially to laterally.

To facilitate handling, the distal and proximal ends of the semitendinosus tendon were stitched. Sutures were introduced from the anterosuperior portal, circumventing the upper two‐third of the subscapular tendon, and then retrieved through the anterosuperior portal. This suture was connected to the graft, which then was pulled through the anterosuperior portal into the joint further encircling the subscapular tendon. A CorkScrew 4.5 mm anchor (Arthrex GmbH) with two pairs of sutures was introduced through the anterior portal on the anterior glenoid rim at 2 o'clock. A sliding knot was used to secure the first end of the transplant to the anterior glenoid rim. At 5 o'clock, a second CorkScrew 4.5 mm anchor was inserted, securing the transplant to the glenoid, thus imitating the anterior labrum. The graft leg positioned on the anterior side of the subscapular tendon was then sutured to the 2 o'clock anchor in slight tension to avoid compression of the subscapular tendon while allowing the tendon to slide inside the sling. Care was taken to avoid overtightening of the sling. To further strengthen the semitendinosus graft fixation, additional suture anchors were inserted. Suture ends and the surplus graft length were cut and removed.

### Open Latarjet procedure

**Figure 5:**
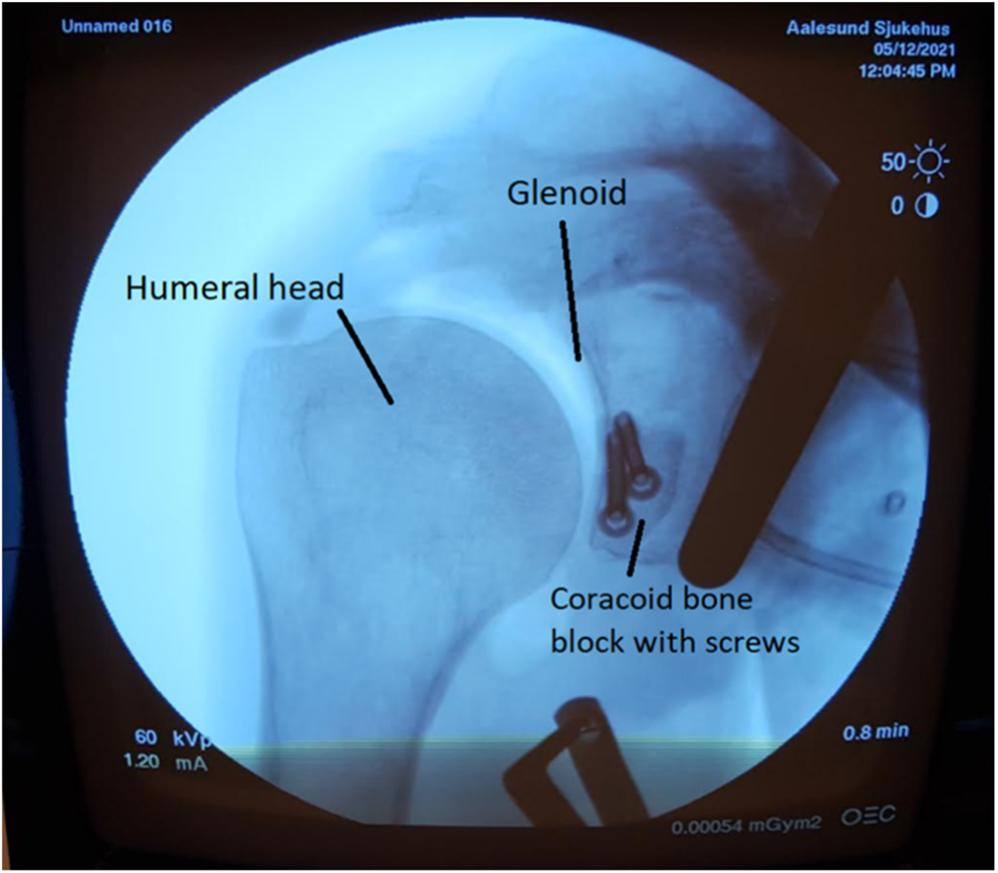
Postoperative X‐ray control with appropriate positioning of the graft.

The SSP was arthroscopically removed and a standard deltopectoral incision from the coracoid and 5 cm toward the axillary fold was performed. The pectoralis minor was released from the coracoid process, and osteotomy of the coracoid was performed with a 90° oscillating saw. Two 2.75 mm drill holes were made 1 cm apart through the coracoid. Through the already‐made incision for the subscapular sling procedure, the coracoid part was fixated to the anterior glenoid with 3.75 mm screws (Arthrex GmbH), after ensuring correct graft position. Correct graft positioning was defined as the flush position in the four finite element models of a glenohumeral joint [23]. The X‐ray control was determined appropriately if the distance between the anterior surface of the graft and the glenoid joint surface's plan was between 5 mm medial and 1 mm lateral which occurred in 100% of the cases. The subscapularis split was left open, and the deltopectoral interval and skin closed. Postoperative X‐ray control with appropriate positioning of the graft (Figure 5).

### Data analysis

Outcomes were analyzed using linear regression models with condition (native, injured, Latarjet, SSP) as the sole covariate. To address the nonindependence of repeated measurements on the same shoulder, cluster robust standard errors (SEs) were applied, with each shoulder serving as a cluster. This method ensures accurate SE estimation and valid inference. Marginal means for each condition were derived from the estimated regression coefficients, providing adjusted outcome values that account for the clustering within shoulders. Pairwise comparisons between conditions were conducted using these marginal means, enabling direct assessment of differences between experimental groups. Statistical significance was defined as *p* < 0.05. All statistical analyses were performed using Stata, ensuring reproducibility and rigor.

## RESULTS

The stability of the glenohumeral joint was evaluated in 16 specimens by measuring glenohumeral translations under various axial loads across four shoulder configurations. To ensure consistency and minimize variability, all procedures were performed by the same two dedicated surgeons, following a standardized protocol. No significant complications were observed during the surgeries, and postoperative radiographs confirmed proper implant and repair placement. The stability outcomes of the SSP were compared against the established Latarjet procedure.

### Anterior loading condition

The SSP demonstrated superior stability over the Latarjet procedure in the neutral (0/0°; *p* < 0.001), external rotation (0/60°; *p* = 0.007) and abduction (60/0°; *p* < 0.001) positions, with significantly reduced glenohumeral translations (Table 1). Stability was comparable between SSP and Latarjet in the abducted and rotated position (60/60°; *p* = 0.379). The SSP also significantly improved stability relative to the injured state with critical glenoid bone loss (*p* = 0.006). In contrast, the Latarjet procedure showed inferior stability in neutral (0/0°; *p* = 0.002) and abducted (60/0°; *p* = 0.014) positions, with destabilizations of 9.5 and 8.9 mm, respectively.

**Table 1:** Anterior axial applied loads: Recorded glenohumeral joint translations for anterior axial applied loads for various shoulder configurations across the four groups.

Comparison	Difference	Standard error	*p* Value	Clinical implication
Anterior 0/0°				
Injured vs. Latarjet	−9.54 mm	2.49 mm	0.002^a^	Latarjet condition shows significantly lower stability
Injured vs. Native	1.46 mm	0.76 mm	0.076	No significant difference in stability
Injured vs. SSP	4.83 mm	2.14 mm	0.040^a^	SSP condition shows significantly better stability
Latarjet vs. Native	11.00 mm	2.29 mm	<0.001^a^	Native condition shows significantly better stability
Latarjet vs. SSP	14.37 mm	2.33 mm	<0.001^a^	SSP condition shows significantly better stability
Native vs. SSP	3.37 mm	2.13 mm	0.136	No significant difference in stability
Anterior 0/60°				
Injured vs. Latarjet	2.16 mm	1.46 mm	0.159	No significant difference in stability
Injured vs. Native	2.82 mm	1.23 mm	0.037^a^	Native condition shows significantly better stability
Injured vs. SSP	5.84 mm	1.83 mm	0.006^a^	SSP condition shows significantly better stability
Latarjet vs. Native	0.65 mm	1.25 mm	0.609	No significant difference in stability
Latarjet vs. SSP	3.67 mm	1.17 mm	0.007^a^	SSP condition shows significantly better stability
Native vs. SSP	3.02 mm	1.65 mm	0.088	Marginal difference; SSP condition may show better stability
Anterior 60/0°				
Injured vs. Latarjet	−8.91 mm	3.22 mm	0.014^a^	Latarjet condition shows significantly lower stability
Injured vs. Native	5.16 mm	1.66 mm	0.007^a^	Native condition shows significantly better stability
Injured vs. SSP	4.25 mm	2.64 mm	0.129	No significant difference in stability
Latarjet vs. Native	14.08 mm	2.40 mm	<0.001^a^	Native condition shows significantly better stability
Latarjet vs. SSP	13.17 mm	2.20 mm	<0.001^a^	SSP condition shows significantly better stability
Native vs. SSP	−0.91 mm	1.46 mm	0.539	No significant difference in stability
Anterior 60/60°				
Injured vs. Latarjet	1.07 mm	1.25 mm	0.408	No significant difference in stability
Injured vs. Native	2.54 mm	1.30 mm	0.071	No significant difference in stability
Injured vs. SSP	1.76 mm	1.29 mm	0.194	No significant difference in stability
Latarjet vs. Native	1.47 mm	0.61 mm	0.030^a^	Native condition shows significantly better stability
Latarjet vs. SSP	0.69 mm	0.766 mm	0.379	No significant difference in stability
Native vs. SSP	−0.77 mm	1.46 mm	0.261	No significant difference in stability

*Note*: Samples in each configuration were subjected to directional loads of 30 N. Results are presented as mean differences, standard errors and *p* Values.

a Indicates a statistically significant difference.

### Anteroinferior loading condition

The SSP outperformed the Latarjet procedure under anteroinferior loading, with significantly lower glenohumeral translations in neutral (0/0°; *p* = 0.003) and 60° abduction (*p* < 0.001) (Table 2). The SSP effectively restored stability in these positions relative to the injured state (*p* < 0.001 for neutral; *p* = 0.026 for abduction). Minimal differences were observed between SSP and native shoulders in the neutral position (mean difference 2.2 mm; *p* = 0.075). The Latarjet procedure did not yield significant stability improvements in any anteroinferior condition.

**Table 2:** Anteroinferior axial applied loads: Recorded glenohumeral joint translations for anteroinferior axial applied loads for various shoulder configurations across the four groups.

Comparison	Difference	Standard error	*p* Value	Clinical implication
Anteroinferior (0/0°)				
Injured vs. Latarjet	0.911 mm	2.08 mm	0.668	No significant difference in stability
Injured vs. Native	3.50 mm	0.58 mm	<0.001^a^	Native condition shows significantly better stability
Injured vs. SSP	5.71 mm	1.067785	<0.001^a^	SSP condition shows significantly better stability
Latarjet vs. Native	2.59 mm	2.054954	0.227	No significant difference in stability
Latarjet vs. SSP	4.80 mm	1.338201	0.003^a^	SSP condition shows significantly better stability
Native vs. SSP	2.21 mm	1.155453	0.075	Marginal difference; SSP condition may show better stability
Anteroinferior (0/60°)				
Injured vs. Latarjet	−0.02 mm	1.84 mm	0.991	No significant difference in stability
Injured vs. Native	4.90 mm	0.83 mm	<0.001^a^	Native condition shows significantly better stability
Injured vs. SSP	−1.06 mm	1.67 mm	0.534	No significant difference in stability
Latarjet vs. Native	4.92 mm	1.83 mm	0.017^a^	Native condition shows significantly better stability
Latarjet vs. SSP	−1.04 m	1.33 mm	0.444	No significant difference in stability
Native vs. SSP	−5.97 mm	1.83 mm	0.005^a^	Native condition shows significantly better stability
Anteroinferior (60/0°)				
Injured vs. Latarjet	−4.34 mm	2.37 mm	0.088	Marginal difference; Latarjet may show lower stability
Injured vs. Native	11.67 mm	1.39 mm	<0.001^a^	Native condition shows significantly better stability
Injured vs. SSP	5.55 mm	2.25 mm	0.026^a^	SSP condition shows significantly better stability
Latarjet vs. Native	16.01 mm	1.52 mm	<0.001^a^	Native condition shows significantly better stability
Latarjet vs. SSP	9.90 mm	1.39 mm	<0.001^a^	SSP condition shows significantly better stability
Native vs. SSP	−6.11 mm	1.63 mm	0.002^a^	Native condition shows significantly better stability
Anteroinferior (60/60°)				
Injured vs. Latarjet	0.45 mm	1.28 mm	0.741	No significant difference in stability
Injured vs. Native	2.94 mm	1.27 mm	0.036^a^	Native condition shows significantly better stability
Injured vs. SSP	−0.76 mm	1.57 mm	0.633	No significant difference in stability
Latarjet vs. Native	2.48 mm	0.55 mm	<0.001^a^	Native condition shows significantly better stability
Latarjet vs. SSP	−1.21 mm	0.88 mm	0.191	No significant difference in stability
Native vs. SSP	−3.70 mm	1.11 mm	0.005^a^	Native condition shows significantly better stability

*Note*: Samples in each configuration were subjected to directional loads of 30 N. Results are presented as mean differences, standard errors and *p* Values.

a Indicates a statistically significant difference.

### Inferior loading condition

Under inferior loading, the SSP showed significant stability improvements over the Latarjet procedure in neutral (0/0°; *p* = 0.005), abduction (60/0°; *p* = 0.02) and rotation (0/60°; *p* = 0.002) positions (Table 3). Stability was similar between the procedures in the abducted and rotated (60/60°) position. The SSP did not fully replicate native shoulder stability, particularly under inferior loading. The Latarjet procedure did not significantly improve stability in any position and led to significant deterioration in abduction (60/0°; *p* = 0.036).

**Table 3:** Inferior axial applied loads: Recorded glenohumeral joint translations for inferior axial applied loads for various shoulder configurations across the four groups.

Comparison	Difference	Standard error	*p* Value	Clinical implication
Inferior 0/0				
Injured vs. Latarjet	−1.12 mm	0.65 mm	0.108	Latarjet condition shows slightly lower stability (nonsignificant; NS)
Injured vs. Native	6.16 mm	1.58 mm	0.001^a^	Native condition shows significantly better stability
Injured vs. SSP	1.21 mm	0.66 mm	0.086	SSP condition shows better stability (NS)
Latarjet vs. Native	7.28 mm	1.62 mm	<0.001^a^	Native condition shows significantly better stability
Latarjet vs. SSP	2.34 mm	0.72 mm	0.005^a^	SSP condition shows significantly better stability
Native vs. SSP	−4.94 mm	1.76 mm	0.014^a^	Native condition shows significantly better stability
Inferior 0/60°				
Injured vs. Latarjet	−2.48 mm	1.48 mm	0.115	Latarjet condition shows lower stability (NS)
Injured vs. Native	7.45 mm	1.17 mm	<0.001^a^	Native condition shows significantly better stability
Injured vs. SSP	2.80 mm	1.49 mm	0.080	SSP condition shows better stability (NS)
Latarjet vs. Native	9.94 mm	1.68 mm	<0.001^a^	Native condition shows significantly better stability
Latarjet vs. SSP	5.29 mm	1.41 mm	0.002^a^	SSP condition shows significantly better stability
Native vs. SSP	−4.65 mm	1.74 mm	0.018	Native condition shows significantly better stability
Inferior 60/0°				
Injured vs. Latarjet	−4.71 mm	2.04 mm	0.036^a^	Latarjet condition shows significantly lower stability
Injured vs. Native	7.15 mm	1.45 mm	<0.001^a^	Native condition shows significantly better stability
Injured vs. SSP	0.52 mm	1.36 mm	0.704	No significant difference in stability
Latarjet vs. Native	11.87 mm	2.21 mm	<0.001^a^	Native condition shows significantly better stability
Latarjet vs. SSP	5.24 mm	1.42 mm	0.002^a^	SSP condition shows significantly better stability
Native vs. SSP	−6.62 mm	1.38 mm	<0.001^a^	Native condition shows significantly better stability
Inferior 60/60°				
Injured vs. Latarjet	−1.52 mm	0.90 mm	0.113	Latarjet condition shows lower stability (NS)
Injured vs. Native	2.34 mm	0.73 mm	0.006^a^	Native condition shows significantly better stability
Injured vs. SSP	−1.11 mm	1.15 mm	0.350	No significant difference in stability
Latarjet vs. Native	3.86 mm	0.95 mm	0.001^a^	Native condition shows significantly better stability
Latarjet vs. SSP	0.40 mm	0.51 mm	0.443	No significant difference in stability
Native vs. SSP	−3.456125	1.146213	0.009^a^	Native condition shows significantly better stability

*Note*: Samples in each configuration were subjected to directional loads of 30 N. Results are presented as mean differences, standard errors and *p* Values.

a Indicates a statistically significant difference.

### ROM assessments

The Latarjet procedure resulted in significantly greater ROM than the SSP in the neutral position (*p* = 0.002), with the SSP's ROM being similar to that of native shoulders but with high variability (SE = 4.67 mm) (Table 4). The Latarjet procedure also produced greater ROM compared to native shoulders (*p* = 0.003). At 60° abduction, the SSP showed significantly higher ROM compared to the native group (*p* = 0.029) and injured group (Table 5; mean ∆ = −32.67 mm; *p* = 0.011). The Latarjet procedure provided greater ROM relative to both the injured and native groups (Table 5), with potential stability trade‐offs.

**Table 4:** External rotation in 0° abduction: Robotically mediated rotation applied to shoulders configured in 0° abduction in assessing range of motion.

Comparison	Mean difference	Standard error	*p* Value	Clinical implications
0° abduction				
Injured vs. Latarjet	−33.10°	13.16°	0.024^a^	Significant increase in range of motion (ROM) with Latarjet
Injured vs. Native	−18.57°	11.68°	0.133	No significant difference, indicating similar ROM
Injured vs. SSP	−16.80°	12.54°	0.200	No significant difference, indicating similar ROM
Latarjet vs. Native	14.52°	4.16°	0.003^a^	Significant increase in ROM with Latarjet
Latarjet vs. SSP	16.29°	4.37°	0.002^a^	Significant increase in ROM with Latarjet
Native vs. SSP	1.77°	4.67°	0.709	No significant difference, indicating similar ROM

*Note*: Results are presented as mean differences, standard errors and *p* Values.

a Indicates a statistically significant difference.

**Table 5:** External rotation in 60° abduction: Robotically mediated external rotation applied to shoulders configured in 60° abduction in assessing range of motion.

Comparison	Mean difference	Standard error	*p* Value	Clinical implications
60° abduction				
Injured vs. Latarjet	−33.10°	13.16°	0.024^a^	Significant increase in range of motion (ROM) with Latarjet compared to injured
Injured vs. Native	−18.57°	11.68°	0.133	No significant difference
Injured vs. SSP	−16.80°	12.54°	0.200	No significant difference
Latarjet vs. Native	14.52°	4.16°	0.003^a^	Significant increase in ROM with Latarjet
Latarjet vs. SSP	16.29°	4.37°	0.002^a^	Significant increase in ROM with Latarjet
Native vs. SSP	1.77°	4.67°	0.709	No significant difference

*Note*: Results are presented as mean differences, standard errors and *p* Values.

a Indicates a statistically significant difference.

## DISCUSSION

The current study is an in vitro biomechanical assessment of a novel SSP, specifically its comparative performance to an open Latarjet procedure. Glenohumeral translations were recorded sequentially across groups for common functional positions and higher‐risk arm positions in a broader analysis being subjected to relevant directional forces. The primary finding in this study is that the novel SSP method exhibited superior stability outcomes in a majority of the tested positions, compared to the open Latarjet procedure.

Under anteroinferior loads, the SSP outperformed the Latarjet group in neutral (0/0°; *p* = 0.003) and abducted (60/0°; *p* < 0.001) positions, with comparable outcomes in the remaining configurations. In these positions, the SSP significantly restored stability compared to stability outcomes recorded in samples under conditions of critical bone loss (*p* < 0.001; *p* = 0.026). However, stability parameters were not significantly different for the injured group in the remaining positions. While the SSP restoration of inferior stability in the neutral position (0/0°) was only marginally different from reference (native) shoulders, it was significantly different in all other positions. The Latarjet procedure did not confer improvements in stability across all conditions.

In anterior directional load, the SSP conferred significant improvements in stability than Latarjet in neutral (0/0°; *p* < 0.001), rotated (0/60°; *p* = 0.007) and abducted shoulder positions (60/0°; *p* < 0.001). Anterior stability was similar in the abducted and rotated shoulder (60/60°; *p* = 0.379). Substantial anterior stability improvements were conferred by the SSP compared to Latarjet, specifically in neutral and abducted positions (60/0°). In terms of restoring stability initially lost in conditions of critical glenoid bone loss, significant improvements occurred in neutral and rotated positions (0/60°; *p* = 0.006). Anterior stability in neutral and 60° abduction and rotation (60/60°) was not significantly different, which may indicate that anterior applied force may not reveal instability with sufficient sensitivity in these positions. Moreover, only in the rotation position (0/60°) was there a marginal difference in anterior stability compared to reference shoulders (*p* = 0.088), while all other tested configurations showed stability similar to native shoulders. In shoulders that were abducted and rotated (60/60°) or just rotated (0/60°), the Latarjet procedure did not show significant differences compared to the injured group (*p* = 0.408; *p* = 0.159, respectively). Additionally, worse stability was observed in neutral (0/0°; *p* = 0.002) and abducted (60/0°; *p* = 0.014) configurations under anterior load, with destabilization measured at 9.5 and 8.9 mm, respectively, compared to injured shoulders.

Inferior loading conditions mirrored the stability outcomes observed for the anterior direction, with the SSP group showing significant improvements in neutral, abduction and rotation positions (0/0°; *p* = 0.005; 60/0°; *p* = 0.02; 0/60°; *p* = 0.002) compared to the Latarjet group. In abduction and rotation (60/60°), the outcomes were similar. Minor, nonsignificant numerical improvements in the SSP group were noted compared to conditions of critical glenoid bone loss (injured group) in the neutral (0/0°; *p* = 0.086) and rotated positions (0/60°; *p* = 0.080), with no meaningful difference in the remaining positions. In no tested position did the SSP restore stability to the level of reference shoulders. Inferior stability did not improve in any of the four positions following the Latarjet procedure and significantly worsened in abduction (60/0°; *p* = 0.036), a pattern not dissimilar to what was observed during anterior directional testing. The SSP has one major advantage over the LP: whereas the graft length of the LP and thus its tension when attached is given, the SSP can be further tightened during positioning and fixation and may explain its observed superiority.

ROM was assessed by subjecting shoulder samples to robot‐mediated external rotation forces in a 0° (neutral) or 60° abduction configuration. ROM was significantly larger for the Latarjet group than for the SSP (*p* = 0.002) in the neutral position. The SSP group demonstrated similar ROM to native shoulders; however, this is claimed with caution due to a large SE (4.67°) compared to the mean difference (1.77°), which suggests substantial variation in the data. Following Latarjet, the ROM was significantly greater than for reference shoulders (*p* = 0.003). Remaining pairwise comparisons cannot be assessed with reasonable confidence due to data noise, but we may state with some certainty that Latarjet had greater ROM than even the injured group. In 60° abduction, ROM was significantly higher (*p* = 0.029) following SSP compared to the reference group. Furthermore, the ROM was significantly greater for the SSP (mean ∆ −32.67°; *p* = 0.011) than the recorded mean ROM for lesioned shoulder samples (injured group). Slight caution may be applied to this specific interpretation due to a high SE. ROM was greater following the Latarjet procedure compared to both injured and native groups. Overall, the SSP demonstrated comparative advantages in this study's testing environment. Testing showed that the SSP yields significantly better multidirectional stability outcomes in more than half of all tested configurations. However, while comparative efficacy was apparent, in terms of restoring and correcting the mean stability deficits seen with critical glenoid bone loss, this was achieved significantly only in about half of the tested positions and configurations. Moreover, most of these cases did not restore stability to the level of the reference shoulders, overall. Notably, the Latarjet procedure generally exhibited unstable outcomes across all directions and tested configurations, with several instances of increased destabilization compared to conditions of critical glenoid bone loss. In summary, ROM testing in the 0° abduction revealed that the mean ROM was similar between the SSP procedure and the native group, while the Latarjet group exhibited significantly greater ROM. However, the high relative standard error (SE) for the SSP group data impacts the reliability of these findings. In the neutral position, we can assert with some confidence that the Latarjet group showed a significantly higher ROM than the reference shoulders. ROM testing in 60° abduction demonstrated a similarly significantly greater ROM for both the SSP and Latarjet groups compared to native. Although data noise complicates a definitive interpretation, it is likely that both SSP and Latarjet procedures result in an ROM increase that may in fact be significant. Across all data sets, the SSP condition displays more consistent results compared to the Latarjet procedure. This may reflect the varying technical challenges each method presents to the surgeon, suggesting that the SSP procedure may offer better reproducibility of stability outcomes.

This study design has inherent limitations. The cadaveric shoulders used were derived from an elderly population, while most cases of recurrent anterior shoulder instability requiring surgery involve younger patients. Consequently, age‐dependent changes in skeletal muscle mass, cartilage thickness, bone mass density and connective tissue could influence the results. In cadavers, the dynamic stabilizing effects of working muscles are absent, significantly impacting overall stability. Additionally, generalizability in cadaveric studies is limited due to the loss of variable scapular dynamics and normal tissue healing. A significant confounder is the potential for false‐positive signals due to tissue fatigue and laxity from repeated manipulation and use throughout the testing protocol. The same shoulders were used in three prior testing groups, and the cumulative effects of repetitive loading and manipulation likely compromised the structural integrity and biomechanical properties of the tissues. This repeated stress may have progressively increased joint laxity and instability, resulting in higher translations during the tests, contributing also to a wider spread in the recorded data.

Shoulder arthroscopy has evolved considerably since its inception more than three decades ago [24]. Management of persistent anterior shoulder instability has advanced through innovations in techniques, instruments and biomaterials. Research has further refined treatment approaches by elucidating key pathoanatomic mechanisms involved in this condition, leading to more sophisticated and effective treatment algorithms. Despite these advancements, critical and subcritical bony defects continue to complicate surgical management of shoulder instability. Soft tissue procedures without augmentation are often inadequate in cases of critical glenoid bone loss, leading to a higher probability of recurrence [3, 36]. Minimally invasive procedures, including Bankart repairs and rotator cuff surgeries, are increasingly preferred [9, 18]. The management of persistent anterior shoulder instability using arthroscopic procedures is expected to see significant improvements. As a heterogeneous condition, it commonly requires individualized treatment plans, which necessitate the availability of technically feasible, effective and safe procedures. Arthroscopic procedures offer additional benefits such as faster recovery, less postoperative pain, and better functional outcomes compared to open procedures [2, 29]. The SSP, as an arthroscopic procedure, is less technically challenging than both open and arthroscopic LP. It avoids complications related to bone procedures, like osteolysis or graft misplacement, and minimizes the risk of injury to adjacent neurovascular structures. An LP revision can still be performed following the SSP if necessary, as the anatomy is preserved [37]. The potentially shorter learning curve of SSP may also make it more accessible to treatment centres with lower patient volumes.

Results from previous biomechanical investigations by the authors suggested that the SSP provides better stability outcomes compared to arthroscopic Bankart repair [37]. The findings of the current study are generally consistent with the authors' past research [19, 20, 37]. While the harvesting of an autograft may be considered a drawback of this procedure, most reported donor site complications have been found to be of minor clinical significance [44]. However, the long‐term clinical implications of surgery involving the subscapular tendon, particularly in terms of muscle biodynamics, are not yet fully understood for the novel SSP method [30].

Overall, the results are encouraging and appear to align reasonably well with prior biomechanical in vitro investigations on the subscapular sling (SSP). Future studies should focus on collecting data to evaluate the long‐term integrity of subscapular sling tissue under repetitive soft tissue loading, particularly in the presence of a critical glenoid defect.

## CONCLUSION

The novel SSP has shown superior stability outcomes compared to the open Latarjet procedure in most shoulder positions and force directions tested. These findings provide further evidence that SSP may be a viable, less invasive alternative to traditional open procedures for managing shoulder instability.

## AUTHOR CONTRIBUTIONS

Terje Vagstad, Jan Arild Klungsøyr, Petter Klungsøyr, Jon Olav Drogset and Erland Hermansen were involved in planning, executing, and drafting the manuscript. Christian Bjerknes and Tor Åge Myklebust contributed with the writing of the manuscript and data analysis. Andreas Dalen and Aleksander Skrede contributed in the surgical procedures and robotic measurements, and also writing of the manuscript.

## CONFLICT OF INTEREST STATEMENT

The authors declare no conflict of interest.

## ETHICS STATEMENT

Committees for Medical and Health Research Ethics in Norway (reference number: 2018/2023 REK sør‐øst).

## Data Availability

Data from the biomechanical study is stored with the corresponding author at Møre and Romsdal Hospital Trust. Data can be available upon reasonable request.
